# Impact of Facility Criteria Revision for Rotational Atherectomy on Outcomes After PCI: A Quasi-Experimental Difference-in-Differences Study

**DOI:** 10.1016/j.jacadv.2026.102672

**Published:** 2026-03-12

**Authors:** Tadao Aikawa, Yuichiro Mori, Toshiki Kuno, Yoshihisa Miyamoto, Yuya Matsue, Shun Kohsaka, Kyohei Yamaji, Ken Kozuma, Tohru Minamino

**Affiliations:** a Department of Cardiovascular Biology and Medicine, Juntendo University Graduate School of Medicine, Tokyo, Japan; b Department of Human Health Sciences, Kyoto University Graduate School of Medicine, Kyoto, Japan; c Division of Cardiology, Beth Israel Deaconess Medical Center, Harvard Medical School, Boston, Massachusetts, USA; d Department of Real-World Evidence, Graduate School of Medicine, The University of Tokyo, Tokyo, Japan; e Department of Cardiology, Keio University School of Medicine, Tokyo, Japan; f Department of Health Data Science, Yokohama City University Graduate School of Data Science, Yokohama, Japan; g Department of Cardiovascular Medicine, Kyoto University Graduate School of Medicine, Kyoto, Japan; h Department of Internal Medicine, Division of Cardiology, Teikyo University School of Medicine, Tokyo, Japan

**Keywords:** coronary atherectomy, difference-in-differences analysis, in-hospital mortality, observed-to-expected event ratio, rotational atherectomy

## Abstract

**Background:**

In April 2020, the facility criteria for using rotational atherectomy (RA) during percutaneous coronary intervention (PCI) were updated in Japan. Board-certified PCI operators are now permitted to perform RA during PCI in low-volume hospitals (<200 PCIs/y) without on-site surgical backup (ie, nontraining facilities).

**Objectives:**

This study investigated the impact of the 2020 RA facility criteria revision on in-hospital PCI outcomes in nontraining facilities.

**Methods:**

The authors conducted a retrospective, time-series analysis of patients who underwent PCI in Japan using the J-PCI (Japanese Percutaneous Coronary Intervention) registry, which covers over 90% of all PCIs performed in Japan. Using a quasi-experimental difference-in-differences analysis, the authors compared changes in PCI outcomes between training and nontraining facilities before (2019-2020) and after (2021-2023) the 2020 RA facility criteria revision. The primary outcome was in-hospital mortality after PCI.

**Results:**

Among the 1,161,862 PCIs performed in 1,243 hospitals from 2019 to 2023, the rate of RA use during PCI increased from 4.2% in 2019 to 5.2% in 2023. Of the 1,181 hospitals performing PCI in 2023, 400 (33.9%) had never performed RA. Difference-in-differences regression analyses revealed a slight upward trend in in-hospital mortality in both training (1.6% to 1.9%) and nontraining (1.7% to 1.9%) facilities after the facility criteria revision. However, these trends did not differ significantly between the 2 groups (adjusted *P* = 0.55 for interaction [facility × time]).

**Conclusions:**

The 2020 RA facility criteria revision in Japan did not significantly increase in-hospital mortality after PCI in low-volume, nontraining facilities without on-site surgical backup that were newly accredited for RA use after the revision.

Coronary artery calcification is associated with an increased risk of stent underexpansion and adverse cardiovascular events after percutaneous coronary intervention (PCI).^1^ Rotational atherectomy (RA) can improve stent delivery and prevent stent underexpansion by debulking coronary calcification,^2^, ^3^, ^4^, ^5^ and it is recommended for heavily calcified lesions as a Class 2a indication in both the U.S. and Japanese guidelines.^6^^,^^7^ The North American Expert Review on RA provides the following recommendations for facilities and operators performing RA: 1) device-specific training; 2) establishing a minimum volume of PCI for operators and facilities to perform RA; 3) introducing a licensing system for RA use to operators and facilities; 4) having sufficient and accessible equipment to respond to RA-related complications; and 5) having on-site cardiac surgery or ensuring immediate availability via interhospital transfer.^5^ Several studies have shown that an increase in hospital and operator RA volume is associated with better in-hospital outcomes after PCI with RA.^8^, ^9^, ^10^

Until April 2020, RA use during PCI was permitted only in training facilities certified by the Japanese Association of Cardiovascular Intervention and Therapeutics (CVIT) in Japan. These training facilities are required to fulfill the following criteria: 1) having board-certified PCI operators; 2) performing an annual minimum volume of 200 PCIs; and 3) performing an annual minimum of 30 open-heart surgeries and/or coronary artery bypass graft surgeries by full-time cardiovascular surgeons on staff (ie, on-site surgical backup). In April 2020, the Japanese Ministry of Health, Labour and Welfare and the CVIT revised the facility criteria for RA use. This revision allows board-certified PCI operators who have completed RA device training to perform RA even at hospitals with low PCI volumes (performing <200 PCIs per year) and without on-site surgical backup (ie, nontraining facilities).^2^ In addition, regular RA device-specific training has become mandatory at hospitals performing ≤10 RAs per 2 years. While there is concern that RA use in these newly accredited nontraining facilities may increase PCI complications,^11^ the impact of the 2020 RA facility criteria revision on overall PCI outcomes in Japan has yet to be verified. Importantly, information on any discernible changes in PCI outcomes after the RA facility criteria revision would be useful for revising the facility criteria for other devices.

In this study, we used a quasi-experimental difference-in-differences approach with a Japanese nationwide PCI registry to examine the impact of the 2020 RA facility criteria revision on in-hospital PCI outcomes in Japan.

## Methods

The data supporting the results of this study are limited in availability because of the confidentiality contract between the CVIT and the data provider (ie, the National Clinical Database of Japan). The data provider will review and decide on each reasonable request for a minimum subset of the data for replicating the analysis. The statistical code is available from the corresponding author.

### National PCI data registry (J-PCI registry) and study population

This study used data from the J-PCI (Japanese Percutaneous Coronary Intervention) registry, a prospective, nationwide, multicenter registry of PCI managed by the CVIT. Information on the clinical characteristics and in-hospital outcomes of patients undergoing PCI is collected from over 1,000 hospitals in Japan, covering more than 90% of all PCIs performed in the country. The J-PCI registry’s design and variable definitions have been previously described in detail.^12^, ^13^, ^14^, ^15^ The Institutional Review Board Committee of the Network for Promotion of Clinical Studies (a specified nonprofit organization affiliated with the Osaka University Graduate School of Medicine [Osaka, Japan]) approved the study protocol of the J-PCI registry in compliance with the Declaration of Helsinki. Due to the retrospective and observational nature of the study, written informed consent was waived.

In Japan, registering PCI cases in the J-PCI registry is a mandatory requirement for the CVIT board certification and renewal. This ensures compliance with device use standards at facilities. As a result, data registration rates and completeness remain high. Since January 2013, the J-PCI registry has been integrated into the National Clinical Database, which is an internet-based nationwide prospective registry. Each hospital must appoint a data manager who is responsible for collecting and entering data into the online database system. To ensure data quality, the CVIT holds annual meetings for data managers and conducts random audits of 20 facilities each year.

**Figure 1:**
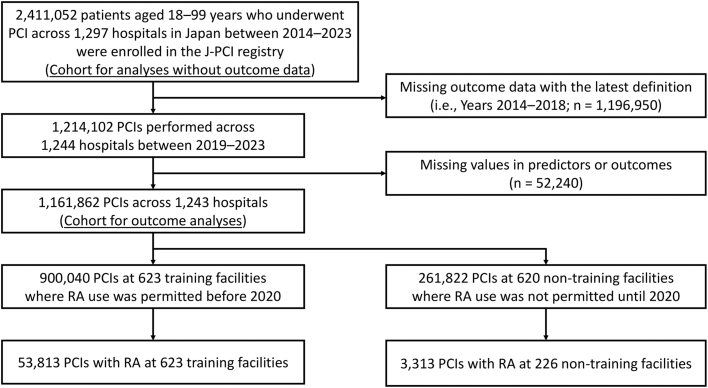
**Flowchart Detailing Patient Selection** PCI = percutaneous coronary intervention; RA = rotational atherectomy.

For the present study, we conducted a retrospective time-series analysis from January 2014 to December 2023. We examined data from 2,411,052 patients between the ages of 18 and 99 who underwent PCI across 1,297 hospitals in Japan to assess temporal trends in RA use during PCI in Japan (Figure 1). Next, we focused on data collected from January 2019 to December 2023 (n = 1,214,102) to analyze the clinical outcomes of PCI with RA. Data from January 2014 to December 2018 (n = 1,196,950) were excluded due to updates in outcome definitions within the J-PCI registry, last revised in January 2019.^12^ PCIs with missing variables for clinical characteristics or outcomes (n = 52,240) were also excluded. Finally, the remaining 1,161,862 PCIs across 1,243 hospitals were included in the cohort for outcome analyses. Of those, 900,040 PCIs (RA use: 53,813 PCIs) were performed in 623 CVIT-certified training facilities, and the remaining 262,822 PCIs (RA use: 3,313 PCIs) were performed in 226 of the 620 nontraining facilities.

### Clinical outcomes

The primary outcome was in-hospital mortality, which was defined as death before hospital discharge or within 30 days after PCI if the patient was hospitalized for more than 30 days. The secondary outcome was a composite of in-hospital death or any complications, including PCI-related myocardial infarction, “definite” stent thrombosis (as defined by the Academic Research Consortium),^16^ cardiac tamponade, cardiogenic shock requiring mechanical and/or inotropic support, emergency surgery, and bleeding requiring a blood transfusion. The J-PCI registry collects in-hospital deaths and complications separately for each patient, which allowed us to avoid double-counting events.

### Statistical analyses

Continuous variables are presented as the mean ± SD. Categorical variables are presented as frequencies and percentages. One-way analysis of variance was used to assess differences between groups for continuous variables, and the chi-square test was used for categorical variables.

We employed a quasi-experimental difference-in-differences regression approach^17^ to compare changes in PCI outcomes between training and nontraining facilities before (years 2019-2020) and after (years 2021-2023) the 2020 RA facility criteria revision. Since April 2020, the revised facility criteria have permitted board-certified PCI operators to perform RA during PCI at nontraining facilities. Training facilities are defined as hospitals with 1) board-certified PCI operators who were qualified by the CVIT as having experience as the primary operator in ≥300 PCI cases and having a high level of knowledge and skills in cardiovascular intervention; 2) an annual hospital PCI volume ≥200 cases; and 3) an annual volume of ≥30 cases of open-heart surgery and/or coronary artery bypass graft surgery performed by full-time cardiovascular surgeons on staff (ie, on-site surgical backup). We adjusted for patient demographics, comorbidities, and preprocedural, procedural, and hospital characteristics (listed in Table 1) in multivariable regression models. We also included an interaction term between the exposure group (training facilities [the control group] vs nontraining facilities [the exposed group]) and time period (preintervention [2019-2020] vs postintervention [2021-2023]) in these models as follows:

Y=α+β1∗(Group)+β2∗(Timeperiod)+β3∗(Group×Timeperiod)+β4∗(Covariates)+ε

where Y represents the outcome variable; α is the outcome in the control group during the preintervention period; β1 is the difference in outcomes between the 2 groups (training vs nontraining facilities) during the preintervention period (years 2019-2020); β2 is the change in outcomes for the training facilities (the control group) from the preintervention to postintervention periods; and ε is the residual error term. The coefficient β3 is the causal parameter of interest, representing the difference-in-differences estimate. This estimate is interpreted as the change in outcomes attributable to the 2020 RA facility criteria revision, which impacted only nontraining facilities.

**Table 1:** Temporal Trends in Patient Characteristics Undergoing PCI in Japan

	Before the Facility Criteria Revision		After the Facility Criteria Revision		
	2019	2020	2021	2022	2023
Age, y					
Training	70.9 ± 11.2	71.1 ± 11.3	71.3 ± 11.3	71.5 ± 11.4	71.7 ± 11.4
Nontraining	71.3 ± 11.2	71.4 ± 11.4	71.6 ± 11.4	71.9 ± 11.4	72.2 ± 11.3
Male					
Training	76.8 (147,313/191,869)	76.8 (139,284/181,365)	76.9 (135,484/176,286)	77.1 (134,171/174,128)	77.1 (136,051/176,392)
Nontraining	75.3 (38,662/51,357)	75.3 (36,757/48,820)	75.5 (38,072/50,421)	75.5 (41,208/54,569)	76.1 (43,101/56,655)
History					
Hypertension					
Training	75.8 (145,496/191,869)	76.4 (138,541/181,365)	76.8 (135,423/176,286)	76.9 (133,969/174,128)	76.5 (134,993/176,392)
Nontraining	73.8 (37,903/51,357)	73.8 (36,050/48,820)	73.8 (37,207/50,421)	74.9 (40,855/54,569)	75.8 (42,966/56,655)
Diabetes					
Training	45.0 (86,281/191,869)	45.6 (82,620/181,365)	45.7 (80,478/176,286)	45.9 (79,946/174,128)	45.7 (80,646/176,392)
Nontraining	43.1 (22,143/51,357)	44.2 (21,595/48,820)	44.5 (22,413/50,421)	45.4 (24,758/54,569)	45.8 (25,932/56,655)
Hyperlipidemia					
Training	67.3 (129,037/191,869)	68.2 (123,660/181,365)	68.6 (120,939/176,286)	68.3 (118,904/174,128)	68.6 (121,050/176,392)
Nontraining	64.4 (33,060/51,357)	65.4 (31,916/48,820)	65.3 (32,907/50,421)	66.1 (36,096/54,569)	66.7 (37,772/56,655)
Dialysis					
Training	7.4 (14,134/191,869)	7.7 (14,036/181,365)	7.9 (13,919/176,286)	7.7 (13,381/174,128)	7.6 (13,384/176,392)
Nontraining	5.2 (2,671/51,357)	5.6 (2,717/48,820)	5.6 (2,839/50,421)	5.7 (3,093/54,569)	5.6 (3,195/56,655)
Chronic lung disease					
Training	2.6 (5,053/191,869)	3.1 (5,652/181,365)	3.3 (5,826/176,286)	3.2 (5,622/174,128)	3.2 (5,699/176,392)
Nontraining	2.7 (1,397/51,357)	2.9 (1,407/48,820)	2.9 (1,472/50,421)	2.9 (1,586/54,569)	3.0 (1,672/56,655)
Peripheral arterial disease					
Training	8.2 (15,793/191,869)	8.7 (15,748/181,365)	8.6 (15,181/176,286)	8.4 (14,571/174,128)	8.1 (14,234/176,392)
Nontraining	6.2 (3,178/51,357)	6.4 (3,115/48,820)	6.3 (3,196/50,421)	6.0 (3,298/54,569)	6.0 (3,382/56,655)
Prior PCI					
Training	46.2 (88,591/191,869)	46.2 (83,737/181,365)	44.0 (77,593/176,286)	44.1 (76,750/174,128)	43.7 (77,144/176,392)
Nontraining	44.0 (22,605/51,357)	43.8 (21,372/48,820)	42.0 (21,198/50,421)	42.8 (23,382/54,569)	43.0 (24,337/56,655)
Prior CABG					
Training	3.6 (6,864/191,869)	3.7 (6,684/181,365)	3.5 (6,211/176,286)	3.3 (5,816/174,128)	3.2 (5,648/176,392)
Nontraining	2.1 (1,100/51,357)	2.2 (1,078/48,820)	2.0 (986/50,421)	2.2 (1,193/54,569)	2.0 (1,159/56,655)
Prior myocardial infarction					
Training	22.6 (43,393/191,869)	23.2 (42,045/181,365)	22.5 (39,583/176,286)	22.9 (39,870/174,128)	22.7 (40,095/176,392)
Nontraining	21.0 (10,798/51,357)	21.2 (10,363/48,820)	20.1 (10,134/50,421)	20.8 (11,345/54,569)	21.1 (11,980/56,655)
Prior heart failure					
Training	15.3 (29,333/191,869)	16.3 (29,551/181,365)	16.6 (29,179/176,286)	16.9 (29,444/174,128)	17.4 (30,743/176,392)
Nontraining	14.2 (7,303/51,357)	14.8 (7,203/48,820)	15.1 (7,618/50,421)	15.9 (8,671/54,569)	17.7 (10,016/56,655)
Preprocedural characteristics					
Elective PCI					
Training	71.9 (138,004/191,869)	71.0 (128,745/181,365)	70.2 (123,834/176,286)	69.3 (120,590/174,128)	68.9 (121,511/176,392)
Nontraining	70.8 (36,366/51,357)	69.2 (33,797/48,820)	69.3 (34,934/50,421)	69.9 (38,150/54,569)	70.0 (39,640/56,655)
Acute coronary syndrome					
Training	37.0 (70,982/191,869)	38.0 (68,898/181,365)	38.7 (68,179/176,286)	39.1 (68,065/174,128)	39.0 (68,876/176,392)
Nontraining	42.0 (21,545/51,357)	43.5 (21,219/48,820)	42.4 (21,361/50,421)	41.3 (22,512/54,569)	41.2 (23,314/56,655)
Cardiac arrest within 24 h					
Training	1.8 (3,495/191,869)	2.0 (3,584/181,365)	2.0 (3,522/176,286)	2.1 (3,679/174,128)	2.2 (3,819/176,392)
Nontraining	1.4 (735/51,357)	1.4 (704/48,820)	1.4 (712/50,421)	1.4 (753/54,569)	1.6 (899/56,655)
Cardiogenic shock within 24 h					
Training	3.3 (6,260/191,869)	3.6 (6,613/181,365)	3.7 (6,477/176,286)	3.9 (6,789/174,128)	4.0 (7,025/176,392)
Nontraining	2.8 (1,462/51,357)	3.1 (1,495/48,820)	3.0 (1,491/50,421)	3.1 (1,668/54,569)	3.2 (1829/56,655)
Acute heart failure within 24 h					
Training	4.0 (7,731/191,869)	4.2 (7,677/181,365)	4.4 (7,698/176,286)	4.5 (7,772/174,128)	4.4 (7,686/176,392)
Nontraining	4.2 (2,177/51,357)	4.3 (2,100/48,820)	4.2 (2,131/50,421)	4.5 (2,434/54,569)	4.4 (2,483/56,655)
Anginal symptom within 1 mo					
Training	72.0 (138,185/191,869)	72.3 (131,074/181,365)	73.9 (130,240/176,286)	72.9 (126,925/174,128)	72.8 (128,445/176,392)
Nontraining	73.6 (37,816/51,357)	74.6 (36,419/48,820)	75.0 (37,826/50,421)	74.0 (40,405/54,569)	74.2 (42,049/56,655)
Preprocedural clopidogrel					
Training	35.7 (68,593/191,869)	36.1 (65,442/181,365)	32.3 (57,006/176,286)	28.1 (48,947/174,128)	25.6 (45,079/176,392)
Nontraining	34.1 (17,504/51,357)	33.9 (16,572/48,820)	31.0 (15,615/50,421)	27.6 (15,050/54,569)	26.3 (14,883/56,655)
Preprocedural potent P2Y12 inhibitors (ticagrelor or prasugrel)					
Training	50.6 (97,120/191,869)	50.9 (92,390/181,365)	55.1 (97,055/176,286)	59.7 (103,917/174,128)	62.9 (110,991/176,392)
Nontraining	48.5 (24,898/51,357)	49.5 (24,188/48,820)	53.9 (27,179/50,421)	58.4 (31,855/54,569)	60.3 (34,150/56,655)
Preprocedural anticoagulants					
Training	7.1 (13,700/191,869)	7.6 (13,759/181,365)	8.1 (14,363/176,286)	8.0 (13,908/174,128)	8.1 (14,310/176,392)
Nontraining	6.7 (3,453/51,357)	6.7 (3,268/48,820)	7.1 (3,590/50,421)	7.3 (4,010/54,569)	7.5 (4,250/56,655)
Procedural characteristics					
Arterial access site					
Femoral					
Training	22.1 (42,498/191,869)	20.7 (37,474/181,365)	20.0 (35,287/176,286)	19.0 (33,136/174,128)	17.9 (31,578/176,392)
Nontraining	17.7 (9,078/51,357)	16.3 (7,962/48,820)	15.9 (8,003/50,421)	15.5 (8,451/54,569)	14.5 (8,240/56,655)
Radial					
Training	72.9 (139,784/191,869)	74.1 (134,419/181,365)	75.1 (132,334/176,286)	76.5 (133,212/174,128)	77.7 (137,063/176,392)
Nontraining	76.8 (39,466/51,357)	78.5 (38,345/48,820)	79.2 (39,922/50,421)	79.6 (43,439/54,569)	80.9 (45,845/56,655)
Others					
Training	5.0 (9,587/191,869)	5.2 (9,472/181,365)	4.9 (8,665/176,286)	4.5 (7,780/174,128)	4.4 (7,751/176,392)
Nontraining	5.5 (2,813/51,357)	5.1 (2,513/48,820)	5.0 (2,496/50,421)	4.9 (2,679/54,569)	4.5 (2,570/56,655)
Mechanical circulatory support during PCI					
Training	3.8 (7,377/191,869)	4.3 (7,792/181,365)	4.7 (8,311/176,286)	5.1 (8,871/174,128)	5.1 (9,023/176,392)
Nontraining	3.1 (1,596/51,357)	3.3 (1,591/48,820)	3.4 (1734/50,421)	3.8 (2086/54,569)	4.0 (2,259/56,655)
Drug-eluting stent use					
Training	82.6 (158,418/191,869)	81.6 (148,003/181,365)	80.8 (142,490/176,286)	79.6 (138,655/174,128)	77.5 (136,782/176,392)
Nontraining	85.1 (43,690/51,357)	84.5 (41,270/48,820)	83.8 (42,250/50,421)	83.1 (45,344/54,569)	80.9 (45,823/56,655)
Bare-metal stent use					
Training	0.4 (678/191,869)	0.2 (401/181,365)	0.2 (313/176,286)	0.2 (394/174,128)	0.2 (292/176,392)
Nontraining	0.3 (173/51,357)	0.1 (61/48,820)	0.1 (54/50,421)	0.1 (69/54,569)	0.2 (129/56,655)
Drug-coated balloon use					
Training	18.5 (35,452/191,869)	20.1 (36,504/181,365)	22.2 (39,087/176,286)	23.7 (41,331/174,128)	27.0 (47,634/176,392)
Nontraining	14.4 (7,406/51,357)	15.6 (7,621/48,820)	17.1 (8,609/50,421)	19.1 (10,440/54,569)	22.6 (12,812/56,655)
Rotational atherectomy use					
Training	5.3 (10,249/191,869)	5.6 (10,115/181,365)	6.6 (11,554/176,286)	6.5 (11,287/174,128)	6.0 (10,608/176,392)
Nontraining	0.0 (0/51,357)	0.0 (0/48,820)	1.2 (594/50,421)	2.1 (1,163/54,569)	2.7 (1,556/56,655)
Orbital atherectomy use					
Training	0.0 (0/191,869)	2.2 (4,032/181,365)	2.7 (4,808/176,286)	2.3 (3,994/174,128)	1.8 (3,147/176,392)
Nontraining	0.0 (0/51,357)	0.1 (34/48,820)	0.2 (111/50,421)	0.7 (401/54,569)	0.8 (432/56,655)

Values are mean ± SD or % (n/N). Until April 2020, PCI operators were not permitted to perform rotational atherectomy during PCI at nontraining facilities in Japan. Annual hospital PCI volumes ≥200 cases and on-site surgical backup are mandatory for training facilities.

CABG = coronary artery bypass grafting; PCI = percutaneous coronary intervention.

Our primary objective was to evaluate the overall impact of the facility criteria revision at the level of the targeted facility category. Although a direct mechanistic link between the facility criteria revision and PCI outcomes is likely absent in facilities that did not implement RA, our difference-in-differences analysis did not exclude facilities that did not adopt RA to avoid selection bias.

A difference-in-differences analysis hinges on 2 assumptions: the parallel trend assumption and the common shock assumption.^18^ The parallel trend assumption requires that the difference in outcomes between the training and nontraining facilities remains constant over time in the absence of treatment. The parallel trend assumption was verified through visual inspection of the outcome trends between the 2 groups during the pretreatment period, as well as through the statistical significance of the interaction term between the group indicator and the pretreatment time trend variable. The common shock assumption requires that trends in outcomes among training and nontraining facilities show similar changes in response to any events other than the intervention of interest (ie, the 2020 RA facility criteria revision). However, this assumption cannot typically be verified using data alone. For instance, the temporal coincidence of the facility criteria revision with the onset of the COVID-19 pandemic likely impacted PCI case mix and outcomes nationwide. Since the pandemic affected both types of hospitals simultaneously, it meets the criteria for a common shock within the difference-in-differences framework. We assumed the validity of the common shock assumption because we were unaware of any other events that occurred concurrently with the facility criteria revision and that would have disproportionately impacted either group.

As a sensitivity analysis, we assessed the observed-to-expected (O/E) event ratios with 95% CIs after PCI in the cohort. We developed a PCI risk prediction model to estimate in-hospital mortality using multivariable logistic regression analysis based on data from training facilities between 2019 and 2020 (before the facility criteria revision). All variables included in the difference-in-differences analysis were used to develop this prediction model.

As an exploratory analysis, a multivariable logistic regression analysis was performed to assess the effect of hospital type (training vs nontraining facilities) on the primary outcome in patients undergoing PCI with RA after the 2020 RA facility criteria revision. Associations between outcomes and covariates were presented as adjusted ORs with 95% CIs.

Statistical analyses were performed using R 4.3.2 (R Foundation for Statistical Computing). For all tests, a 2-sided *P* < 0.05 was considered statistically significant.

## Results

### Temporal trends and hospital variations in the use of RA during PCI in Japan

**Figure 2:**
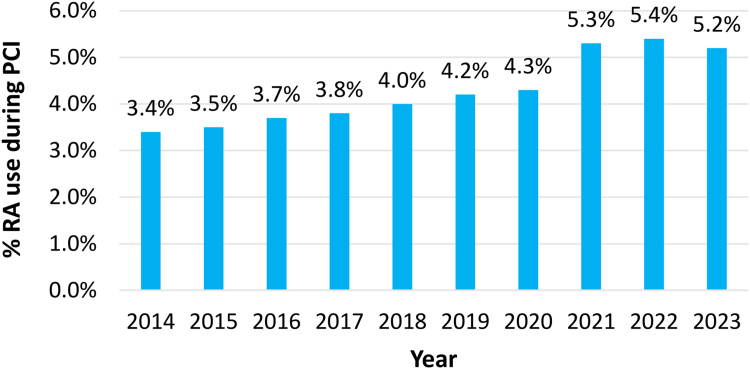
**Temporal Trends in Rotational Atherectomy Use During PCI in Japan** Abbreviations as in Figure 1.

Temporal trends in RA use during PCI in Japan are shown in Figure 2. The rate of RA use during PCI gradually increased from 3.4% (7,402 out of 215,513 PCIs) in 2014 to 4.2% (10,693 out of 253,152 PCIs) in 2019. After the 2020 RA facility criteria revision, the RA use rate increased further, reaching 5.3% (12,902 out of 241,606 PCIs) in 2021 and 5.2% (12,476 out of 242,092 PCIs) in 2023.

**Figure 3:**
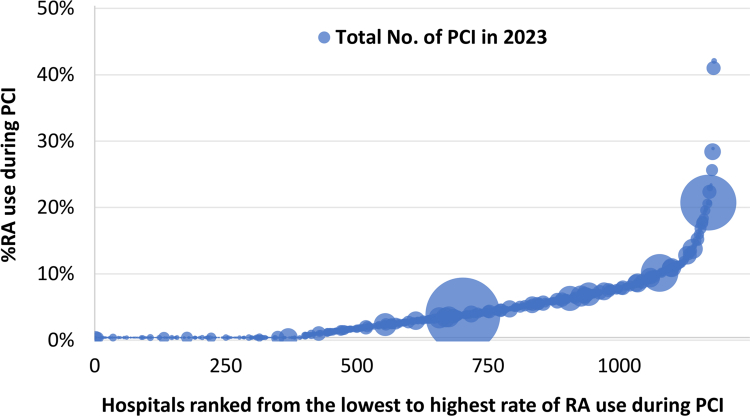
**Variation in Rotational Atherectomy Use Rates by Hospital in 2023** This bubble chart illustrates the variation in the use of RA during PCI at 1,181 Japanese hospitals in 2023. The diameter of each bubble represents the number of PCIs performed at each hospital in 2023. Abbreviations as in Figure 1.

In 2023, RA was performed during PCI at 781 out of 1,181 hospitals. There was significant variation in the RA use rate among hospitals that performed at least one RA procedure in 2023 (median: 4.3%; IQR: 2.3% to 7.2%; maximum: 41.7%; Figure 3).

Temporal trends in the use of drug-eluting stents and drug-coated balloons after RA in Japan are shown in Supplemental Figure 1. Drug-coated balloons have been covered by the Japanese public health insurance and have been clinically available since 2014. The use of drug-eluting stents after RA was prevalent but decreased from 82.7% in 2014 to 67.3% in 2023. Conversely, the use of drug-coated balloons after RA increased from 5.3% in 2014 to 40.9% in 2023.

### Temporal trends in patient characteristics and in-hospital PCI outcomes

The temporal trends in baseline characteristics of patients who underwent PCI in Japan between 2019 and 2023 are shown in Table 1. The mean patient age gradually increased in both types of hospitals, rising from 70.9 years in 2019 to 71.7 years in 2023 in training facilities and from 71.3 years in 2019 to 72.2 years in 2023 in nontraining facilities. The percentage of PCIs performed for acute coronary syndrome gradually increased in training facilities, rising from 37.0% in 2019 to 39.0% in 2023. Conversely, the percentage of elective PCIs gradually decreased in training facilities, dropping from 71.9% in 2019 to 68.9% in 2023. During this period, the use of preprocedural potent P2Y12 inhibitors, preprocedural anticoagulants, and transradial artery access increased in both types of hospitals. In nontraining facilities, the use of RA during PCI increased from 1.2% (594 out of 50,421 PCIs) in 2021 to 2.7% (1,556 out of 56,655 PCIs) in 2023.

The unadjusted temporal trends of in-hospital PCI outcomes in both types of hospitals between 2019 and 2023 are summarized in Table 2. In-hospital mortality after PCI gradually increased in both types of hospitals, rising from 1.6% (2,998 out of 191,869 PCIs) in 2019 to 1.9% (3,439 out of 176,392 PCIs) in 2023 in training facilities, and from 1.6% (830 out of 51,357 PCIs) in 2019 to 1.9% (1,068 out of 56,655 PCIs) in 2023 in nontraining facilities. The incidence of PCI-related myocardial infarction numerically increased over time only in training facilities, rising from 0.5% in 2019 to 0.7% in 2023. The incidence of in-hospital death or any complication after PCI gradually increased in training facilities, rising from 3.1% in 2019 to 3.7% in 2023; this increase paralleled the rise in the proportion of acute coronary syndrome. The results of the tests for the parallel trend assumption are presented in Supplemental Figure 2. Baseline trends in the primary and secondary outcomes did not differ significantly between training and nontraining facilities (adjusted *P* = 0.12 and *P* = 0.42 for the interaction between facility type and time period, respectively).

**Table 2:** Temporal Trends in In-Hospital PCI Outcomes in Japan

	Before the Facility Criteria Revision		After the Facility Criteria Revision		
	2019	2020	2021	2022	2023
In-hospital death					
Training	1.6 (2,998/191,869)	1.7 (3,136/181,365)	1.7 (3,067/176,286)	2.0 (3,444/174,128)	1.9 (3,439/176,392)
Nontraining	1.6 (830/51,357)	1.8 (896/48,820)	1.9 (968/50,421)	1.9 (1,048/54,569)	1.9 (1,068/56,655)
PCI-related myocardial infarction					
Training	0.5 (1,036/191,869)	0.6 (1,135/181,365)	0.7 (1,152/176,286)	0.7 (1,249/174,128)	0.7 (1,246/176,392)
Nontraining	0.5 (277/51,357)	0.5 (235/48,820)	0.5 (254/50,421)	0.6 (304/54,569)	0.5 (311/56,655)
Cardiac tamponade					
Training	0.2 (324/191,869)	0.2 (278/181,365)	0.2 (272/176,286)	0.2 (284/174,128)	0.2 (294/176,392)
Nontraining	0.1 (69/51,357)	0.1 (52/48,820)	0.1 (60/50,421)	0.1 (50/54,569)	0.1 (72/56,655)
Cardiogenic shock requiring mechanical and/or inotropic support					
Training	0.8 (1,584/191,869)	1.0 (1741/181,365)	1.0 (1764/176,286)	1.0 (1720/174,128)	1.0 (1,688/176,392)
Nontraining	0.9 (444/51,357)	0.9 (434/48,820)	0.9 (444/50,421)	0.8 (453/54,569)	0.8 (478/56,655)
Stent thrombosis					
Training	0.1 (255/191,869)	0.1 (245/181,365)	0.1 (228/176,286)	0.1 (228/174,128)	0.1 (217/176,392)
Nontraining	0.2 (89/51,357)	0.2 (80/48,820)	0.2 (83/50,421)	0.1 (73/54,569)	0.1 (66/56,655)
Requirement for emergency surgery					
Training	0.1 (189/191,869)	0.1 (149/181,365)	0.1 (165/176,286)	0.1 (127/174,128)	0.1 (158/176,392)
Nontraining	0.1 (46/51,357)	0.1 (28/48,820)	0.1 (32/50,421)	0.1 (41/54,569)	0.1 (33/56,655)
Bleeding requiring blood transfusion					
Training	0.3 (661/191,869)	0.4 (760/181,365)	0.4 (788/176,286)	0.4 (710/174,128)	0.4 (767/176,392)
Nontraining	0.4 (182/51,357)	0.3 (133/48,820)	0.3 (143/50,421)	0.3 (146/54,569)	0.3 (152/56,655)
Access site bleeding					
Training	0.2 (353/191,869)	0.2 (419/181,365)	0.2 (418/176,286)	0.2 (389/174,128)	0.2 (423/176,392)
Nontraining	0.2 (112/51,357)	0.1 (70/48,820)	0.2 (80/50,421)	0.2 (95/54,569)	0.2 (89/56,655)
Nonaccess site bleeding					
Training	0.2 (321/191,869)	0.2 (358/181,365)	0.2 (385/176,286)	0.2 (331/174,128)	0.2 (366/176,392)
Nontraining	0.1 (71/51,357)	0.1 (64/48,820)	0.1 (66/50,421)	0.1 (54/54,569)	0.1 (63/56,655)
In-hospital death or any complication					
Training	3.1 (5,880/191,869)	3.4 (6,187/181,365)	3.5 (6,214/176,286)	3.8 (6,568/174,128)	3.7 (6,552/176,392)
Nontraining	3.2 (1,624/51,357)	3.3 (1,604/48,820)	3.3 (1,684/50,421)	3.3 (1818/54,569)	3.3 (1863/56,655)

Values are % (n/N). Until April 2020, PCI operators were not permitted to perform rotational atherectomy during PCI at nontraining facilities in Japan. Annual hospital PCI volumes ≥200 cases and on-site surgical backup are mandatory for training facilities.

Abbreviation as in Table 1.

### Temporal trends of in-hospital outcomes in patients undergoing PCI before and after the 2020 RA facility criteria revision

**Figure 4:**
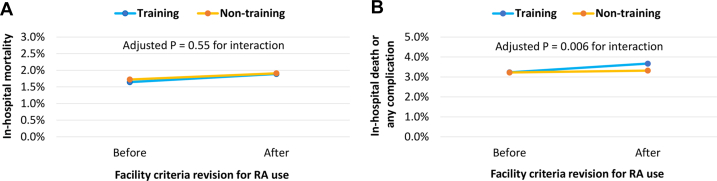
**In-Hospital PCI Outcomes Before and After the RA Facility Criteria Revision** Changes in the primary (A, in-hospital death) and secondary (B, in-hospital death or any complication) outcomes before (years 2019-2020) and after (years 2021-2023) the 2020 facility criteria revision for RA use in training and nontraining facilities are presented. Abbreviations as in Figure 1.

The primary outcome trend (in-hospital death) and the secondary outcome trend (in-hospital death or any complication) before (years 2019-2020) and after (years 2021-2023) the 2020 RA facility criteria revision are presented in Figure 4. Crude in-hospital mortality trended slightly upward in both training (1.6% to 1.9%) and nontraining (1.7% to 1.9%) facilities after the facility criteria revision (Figure 4A). However, these trends did not differ significantly between the 2 groups (adjusted *P* = 0.55 for the interaction between facility type and time period). Similarly, the crude in-hospital death or any complication also trended slightly upward in training (3.2% to 3.7%) and nontraining facilities (3.2% to 3.3%) after the facility criteria revision (Figure 4B). However, this trend increased more in training facilities (adjusted *P* = 0.006 for the interaction between facility type and time period). The details of the adjusted ORs relating to the difference-in-differences analyses of the primary and secondary outcomes are shown in Supplemental Table 1.

### Sensitivity analysis

**Figure 5:**
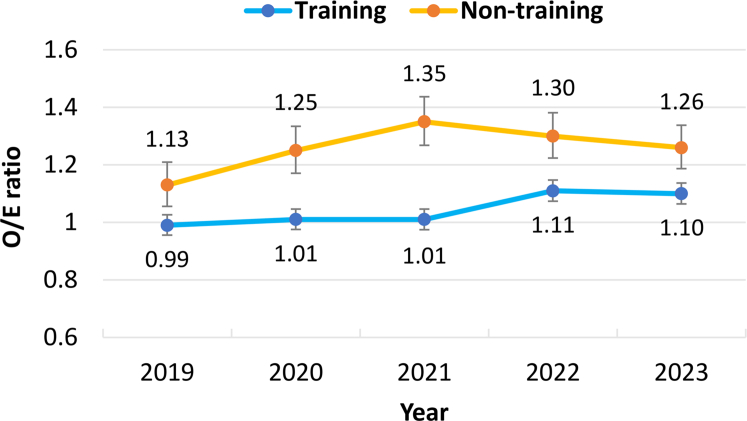
**Temporal Trends of Observed-to-Expected Ratios for In-Hospital Mortality After PCI** The data for each time point are the yearly observed-to-expected (O/E) ratio from January to December. The black dashed line indicates the implementation of the revised facility criteria for RA use in April 2020. The vertical bars represent 95% CIs. Abbreviations as in Figure 1.

The temporal trends of the adjusted O/E ratios for in-hospital death after PCI in both types of facilities are presented in Figure 5. The O/E ratios in nontraining facilities gradually increased from 2019 (1.13; 95% CI: 1.05-1.20) to 2021(1.35; 95% CI: 1.26-1.43), then slightly decreased until 2023 (1.26; 95% CI: 1.19-1.34). In contrast, the O/E ratios in training facilities remained relatively constant from 2019 (0.99; 95% CI: 0.95-1.02) to 2021 (1.01; 95% CI: 0.98-1.05) and steadily increased until 2023 (1.10; 95% CI: 1.06-1.13).

Adjusted ORs for in-hospital mortality in patients undergoing PCI with RA after the 2020 RA facility criteria revision are summarized in Supplemental Table 2. RA performed at training facilities was significantly associated with a lower in-hospital mortality after PCI than RA performed at nontraining facilities that started performing RA after the facility criteria revision (adjusted OR: 0.66; 95% CI: 0.50-0.89; *P* = 0.005). The unadjusted temporal trends of in-hospital outcomes after PCI with RA in both types of hospitals are summarized in Supplemental Table 3.

## Discussion

Using the Japanese nationwide J-PCI registry, we conducted difference-in-differences and O/E ratio analyses and found that the 2020 RA facility criteria revision in Japan did not significantly increase in-hospital mortality after PCI in nontraining facilities that were newly accredited by the CVIT for RA use (Central Illustration). These results should reassure policymakers because they indicate that the 2020 RA facility criteria revision successfully achieved its primary objective of enabling PCI operators at hospitals with low PCI volumes and without on-site surgical backup to perform RA without significantly worsening in-hospital PCI outcomes. We also found that the overall rate of RA use during PCI gradually increased; however, as of 2023, there was significant hospital variation in RA use during PCI among Japanese hospitals.

**Central Illustration:**
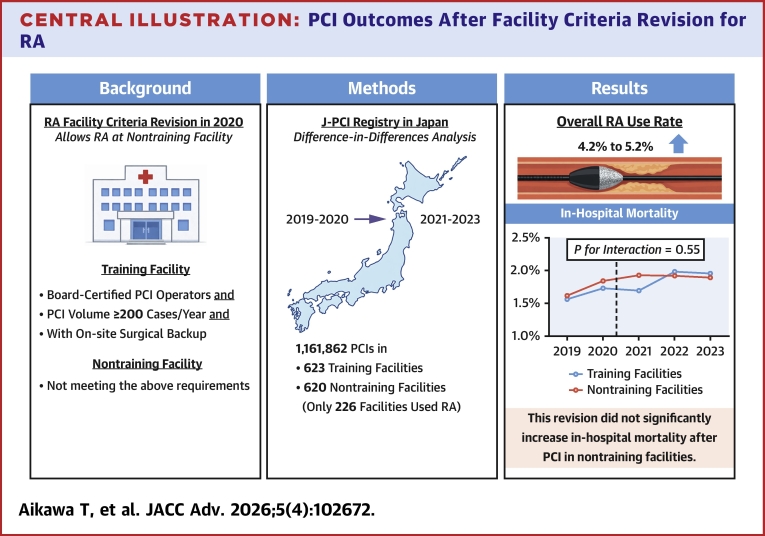
**PCI Outcomes After Facility Criteria Revision for RA** J-PCI = Japanese Percutaneous Coronary Intervention; PCI = percutaneous coronary intervention; RA = rotational atherectomy.

Our difference-in-differences and O/E ratio analyses revealed that the 2020 RA facility criteria revision did not significantly increase in-hospital mortality after PCI in nontraining facilities that began performing RA after the revision. In cases involving moderate or severe calcification, recent expert consensus documents recommend aggressive lesion preparation using RA when: 1) a small balloon or intracoronary imaging catheter cannot cross the target lesion; 2) optimal lesion preparation is not achieved after balloon predilatation; or 3) the target lesion exhibits a large calcified plaque with a maximum angle of >180°, a calcium length of >5 mm, and a calcium thickness of >0.5 mm on intracoronary imaging.^2^^,^^3^^,^^19^ However, RA-related complications, such as slow-flow/no-reflow, coronary perforation/rupture, and burr entrapment, can lead to cardiac tamponade, emergent surgery, and death.^2^^,^^3^^,^^20^ These complications have long been a concern for many PCI operators, particularly at hospitals with low RA volumes^8^^,^^9^ and without on-site surgical backup. Although the use of RA during PCI consistently increased in nontraining facilities after the 2020 RA facility criteria revision, its use remained low at 2.7% in 2023. These data suggest that the 2020 RA facility criteria revision did not cause sufficient changes in RA indications to impact overall PCI outcomes in Japan.

Our study found that RA use during PCI was low but gradually increased up to 5.2% (12,476 out of 242,092 PCIs) in 2023. This trend is similar to that in the United States^9^; however, PCI with atherectomy, including RA, still accounts for only 1% to 3% of all PCI procedures worldwide.^4^^,^^9^ Furthermore, the rate of RA use during PCI varies greatly among institutions, as shown in our study and others.^4^^,^^9^^,^^15^^,^^21^ Since PCI with RA is a technically challenging procedure, several factors are essential to achieving procedural success and optimal patient outcomes. These factors include optimal patient and lesion selection, technical tips for performing RA, and bail-out procedures for RA-related complications.^2^^,^^5^ Furthermore, achieving excellent RA outcomes is expected to depend not only on the experience and skills of PCI operators but also on the preparation and teamwork of the multidisciplinary heart team, including catheterization lab staff and surgeons.^5^ We found that the RA facility criteria revision did not significantly increase in-hospital mortality after PCI at low PCI volume hospitals without on-site surgical backup (ie, nontraining facilities). However, these hospitals exhibited higher O/E ratios for in-hospital death after RA than high PCI volume hospitals with on-site surgical backup (ie, training facilities). High-volume hospitals typically have more staff and experience, leading to better treatment and care (the practice-makes-perfect hypothesis). To improve PCI outcomes by promoting RA use, enhancing the educational infrastructure of heart teams, particularly in hospitals with low PCI or RA volumes, may be essential.

The secondary composite endpoint of procedural complications demonstrated a significant interaction between the hospital type and time. Unadjusted temporal trends of in-hospital outcomes showed that the incidence of PCI-related myocardial infarction slightly increased over time at training facilities while remaining relatively stable at nontraining facilities. Concurrently, the use of atherectomy devices during PCI increased at training facilities over time. Together, these findings suggest that high-volume training facilities may have increasingly performed complex PCI procedures requiring aggressive lesion preparation and the use of atherectomy devices. While these approaches are crucial for treating severely calcified lesions, they may be associated with an increased risk of periprocedural myocardial injury, which manifests as PCI-related myocardial infarction. Importantly, these periprocedural events may not translate into increased short-term mortality, particularly in experienced facilities with well-established procedural protocols and periprocedural management. This may explain why the primary endpoint of in-hospital mortality remained unaffected, whereas the secondary endpoint capturing periprocedural complications demonstrated a significant interaction effect. Overall, these findings suggest that periprocedural complication rates may reflect increasing procedural complexity rather than a deterioration in overall procedural safety.

The relatively low rate of RA use may attenuate the impact of the facility criteria revision when evaluating all PCI cases. However, a standard difference-in-differences analysis restricted to the RA-specific cohort is structurally impossible because RA was prohibited in nontraining facilities prior to the facility criteria revision. Consequently, there are zero RA cases in the control group during the pre-period. Furthermore, from a clinical and epidemiological perspective, our primary objective was to assess the net safety impact of the facility criteria revision on overall PCI outcomes at the facility level rather than the procedural safety of RA itself. The availability of RA may influence operator decision-making for all complex PCI cases, including those in which RA is ultimately not utilized. Restricting the analysis to RA cases alone would introduce substantial selection bias related to lesion characteristics, operator judgment, and institutional practice patterns, none of which are fully captured in the registry. Accordingly, our difference-in-differences analysis should be interpreted as estimating a policy effect similar to an intention-to-treat analysis, capturing the aggregate consequences of the policy change, regardless of whether individual facilities adopted RA after the policy change.

To provide additional context, we conducted the exploratory analysis limited to patients undergoing RA during the postrevision period (Supplemental Table 2). This analysis demonstrated that training facilities were associated with lower in-hospital mortality compared to nontraining facilities (adjusted OR: 0.66; 95% CI: 0.50-0.89; *P* = 0.005). These findings should be interpreted cautiously and are presented as exploratory, given the inherent limitations of subcohort comparisons and the inability to empirically establish a mediation pathway linking changes in RA practice to PCI outcomes.

Importantly, our analysis was not designed to formally test whether changes in RA practice mediated the relationship between the 2020 RA facility criteria revision and PCI outcomes. Instead, changes in RA practice are discussed as a clinically plausible explanatory mechanism rather than a proven causal pathway. Overall, this approach allows us to evaluate the policy’s real-world safety implications while avoiding overinterpretation of mechanistic causality.

### Policy implications

Our study has important policy implications. First, we found that many low-volume hospitals did not immediately initiate RA use during PCI after the 2020 RA facility criteria revision. Additionally, approximately one-third of PCI facilities in Japan did not perform RA in 2023. While the absence of on-site surgical backup is considered a contributing factor, our findings suggest that significant barriers to initiating RA use during PCI persist in nontraining facilities. Since PCI performed without on-site surgical backup has been shown to have similar clinical outcomes to PCI performed with on-site surgical backup,^22^ it is crucial to understand and eliminate these barriers for the long-term success of the revised facility criteria. Further investigation is necessary to determine why RA treatment was not initiated in nontraining facilities, particularly from the perspective of promoting RA use to optimize lesion preparation and stent implantation in severely calcified coronary lesions.

Second, consistent differences in in-hospital mortality rates were observed between training and nontraining facilities when the O/E ratios after RA treatment were compared. This difference is primarily attributed to the number of PCI cases, as several studies, including the J-PCI study, have demonstrated a lower incidence of PCI-related complications in hospitals with high PCI volumes.^13^^,^^23^^,^^24^ Following the 2020 RA facility criteria revision, regular RA device-specific training has been mandated for hospitals with ≤10 RAs per 2 years. To reduce complications following RA use, the effectiveness of this regular device-specific training program in hospitals with low RA volumes must be further evaluated.

### Study limitations

First, although we used a quasi-experimental difference-in-differences regression approach to account for unmeasured confounders, the results rely on several assumptions. In particular, we cannot definitively rule out the possibility that unobserved events concurrent with the facility criteria revision disproportionately influenced on outcomes in hospitals without on-site surgical backup. We also cannot exclude the possibility of differential local effects of the COVID-19 pandemic across hospitals. Second, our results are subject to confounding by indication^25^ because our database lacks the necessary information to evaluate the appropriateness of the RA indication. This information includes lesion morphology, such as the degree of coronary calcification, lesion length, and the presence of bifurcation disease. Nevertheless, intracoronary imaging devices, such as intravascular ultrasound and optical coherence tomography, are commonly used in PCI procedures in Japan,^26^, ^27^, ^28^ suggesting that this patient population was more likely to undergo intravascular imaging during PCI. Third, orbital atherectomy and coronary intravascular lithotripsy are clinically available coronary atherectomy devices that may have influenced the results of this study. RA has been covered by health insurance and used clinically in Japan since 1998, while orbital atherectomy has been available clinically in Japan since 2019. As of 2023, orbital atherectomy was used in only 1.5% of PCIs in Japan (Table 1), suggesting that its impact on this study was minimal. Coronary intravascular lithotripsy, which became available in Japan in December 2022, was not included in the J-PCI registry data through 2023. Both devices are available in Japan under the same facility criteria as RA. Fourth, some patients who underwent PCI with RA at training facilities before the 2020 facility criteria revision may have been transferred from nontraining facilities. The J-PCI registry lacks patient-level identifiers to track interhospital transfers, and this potential referral bias may have affected the results. Reassuringly, RA volumes at training facilities remained stable throughout the study periods (Table 1); however, the residual cannot be excluded. Fifth, the current exposure definition could introduce a transition period in 2020 during which the exposure status would be partially mixed. The J-PCI registry has the exact date of each PCI performed during the study period. However, since cold temperature exposure is a well-described trigger for cardiovascular events,^29^ we defined the preintervention and postintervention periods annually to minimize the impact of seasonal factors. Furthermore, this potential contamination would be expected to bias the estimates toward the null, making our findings conservative. Sixth, although patient outcomes may be correlated within hospitals, fitting generalized linear mixed models with hospital-specific random intercepts was not feasible due to the low event rate and the large proportion of hospitals with zero events during the study period. In such settings, mixed-effects models may fail to converge or produce unstable estimates. Ignoring clustering within hospitals would be expected to underestimate standard errors and inflate type I error. Since our primary analyses using fixed-effects models demonstrated a null result for in-hospital mortality, accounting for clustering with random-effects modeling is unlikely to alter the interpretation of our findings. Finally, while the COVID-19 pandemic overlapped with the policy period, its system-wide impact would be expected to affect both training and nontraining facilities; nonetheless, we cannot fully exclude differential effects.

## Conclusions

In summary, the 2020 RA facility criteria revision in Japan did not significantly increase in-hospital mortality after PCI in low-volume, nontraining facilities without on-site surgical backup that were newly accredited for RA use after the facility criteria revision.

Perspectives **COMPETENCY IN PATIENT CARE AND PROCEDURAL SKILLS:** The North American Expert Review of RA lists the following recommendations for facilities and operators performing RA: 1) device-specific training; 2) establishing a minimum volume of PCI for operators and facilities to perform RA; 3) introducing a licensing system for RA use to operators and facilities; 4) sufficient, accessible equipment to respond to RA-related complications; and 5) on-site cardiac surgery or immediate availability via interhospital transfer. The 2020 facility criteria revision for RA use in Japan, which have permitted board-certified PCI operators to perform RA during PCI at low-volume hospitals (<200 PCIs/y) without on-site surgical backup (ie, nontraining facilities), did not significantly increase in-hospital mortality after PCI in nontraining facilities.

**TRANSLATIONAL OUTLOOK:** Although RA use during PCI consistently increased in Japan, its use remained low at 5.2% in 2023. Further investigation is necessary to determine why RA treatment was not initiated at low-volume hospitals without on-site surgical backup after the 2020 RA facility criteria revision.

## Funding support and author disclosures

This work was supported by the Terumo Life Science Foundation to Dr Aikawa. Dr Aikawa is supported by a Grant-in-Aid for Scientific Research (C) (grant number 25K10897) from the Japan Society for the Promotion of Science. Dr Matsue has received honoraria from Otsuka Pharmaceutical Co, EN Otsuka Pharmaceutical Co Ltd, Novartis Pharma K.K., 10.13039/100013711Bayer Inc, and 10.13039/100004325AstraZeneca; and a collaborative research grant from 10.13039/100010793Pfizer Japan Inc, Otsuka Pharmaceutical Co, EN Otsuka Pharmaceutical Co Ltd, Nippon Boehringer Ingelheim Co Ltd, Roche Diagnostics International Ltd, and Roche Diagnostics K.K. Dr Kozuma has received honoraria from 10.13039/100016242Boston Scientific, Abbott Medical, 10.13039/100004374Medtronic, 10.13039/100019120Otsuka, Daiichi-Sankyo, 10.13039/100002429Amgen, 10.13039/100004336Novartis, Boehringer, 10.13039/100004326Bayer, Life Science Institute, Mochida, and Novo Nordisk Pharma; and has received a Scholarship fund from Abbott Medical. All other authors have reported that they have no relationships relevant to the contents of this paper to disclose.
