# Supplementary material for: Impact of Facility Criteria Revision for Rotational Atherectomy on Outcomes After PCI: A Quasi-Experimental Difference-in-Differences Study
Source: JACC Adv. 2026 Mar 12;5(4):102672. doi: 10.1016/j.jacadv.2026.102672 (PMC12995891; doi:10.1016/j.jacadv.2026.102672)
Supplement: Supplementary Material [file mmc1.docx]

**Supplemental Figure 1.** Trends in the use of drug-eluting stent (DES) or drug-coated balloon (DCB) after rotational atherectomy (RA) in Japan.

**Supplemental Figure 2.** Tests of parallel trend assumption the for the primary outcome (**A**, in-hospital mortality) and the secondary outcome (**B**, in-hospital death or any complication) outcomes between 2019 and 2020.

**Supplemental Table 1.** Adjusted odds ratios in relation to difference-in-differences analyses of the primary outcome (in-hospital death) and the secondary outcome (in-hospital death or any complication) in training and non-training facilities before and after the 2020 facility criteria revision for the use of rotational atherectomy.

|  | **In-hospital death** |  | **In-hospital death or any complication** |  |
| --- | --- | --- | --- | --- |
| **Variables** | **Odds ratio (95% CI)** | **P value** | **Odds ratio (95%CI)** | **P value** |
| Age, per 1 y | 1.060 (1.058-1.061) | <0.001 | 1.03 (1.03-1.03) | <0.001 |
| Male | 0.86 (0.83-0.89) | <0.001 | 0.78 (0.76-0.80) | <0.001 |
| Hypertension | 0.80 (0.77-0.83) | <0.001 | 0.91 (0.89-0.94) | <0.001 |
| Diabetes | 1.17 (1.14-1.21) | <0.001 | 1.02 (0.99-1.04) | 0.13 |
| Hyperlipidemia | 0.63 (0.61-0.65) | <0.001 | 0.84 (0.82-0.86) | <0.001 |
| Dialysis | 1.88 (1.77-1.99) | <0.001 | 1.41 (1.35-1.47) | <0.001 |
| Chronic lung disease | 1.37 (1.27-1.48) | <0.001 | 1.27 (1.20-1.34) | <0.001 |
| Peripheral arterial disease | 1.39 (1.31-1.47) | <0.001 | 1.33 (1.28-1.38) | <0.001 |
| Prior PCI | 0.84 (0.80-0.88) | <0.001 | 0.86 (0.83-0.89) | <0.001 |
| Prior CABG | 1.16 (1.07-1.27) | <0.001 | 1.09 (1.03-1.16) | 0.004 |
| Prior myocardial infarction | 1.15 (1.09-1.21) | <0.001 | 1.24 (1.20-1.28) | <0.001 |
| Prior heart failure | 1.58 (1.51-1.64) | <0.001 | 1.27 (1.23-1.31) | <0.001 |
| Elective PCI | 0.32 (0.30-0.34) | <0.001 | 0.63 (0.61-0.66) | <0.001 |
| Acute coronary syndrome | 2.74 (2.52-2.97) | <0.001 | 1.36 (1.30-1.42) | <0.001 |
| Cardiac arrest within 24 h | 2.57 (2.44-2.71) | <0.001 | 1.97 (1.88-2.07) | <0.001 |
| Cardiogenic shock within 24 h | 2.50 (2.38-2.62) | <0.001 | 2.09 (2.00-2.17) | <0.001 |
| Acute heart failure within 24 h | 1.85 (1.78-1.93) | <0.001 | 1.59 (1.53-1.65) | <0.001 |
| Anginal symptom within 1 month | 0.57 (0.53-0.62) | <0.001 | 0.89 (0.86-0.93) | <0.001 |
| Pre-procedural clopidogrel | 0.80 (0.76-0.84) | <0.001 | 0.89 (0.86-0.92) | <0.001 |
| Pre-procedural potent P2Y12 inhibitors (ticagrelor or prasugrel) | 0.84 (0.80-0.87) | <0.001 | 0.95 (0.92-0.98) | <0.001 |
| Pre-procedural anticoagulants | 1.03 (0.97-1.10) | 0.33 | 1.05 (1.01-1.10) | 0.017 |
| Arterial access site (vs. femoral) |  |  |  |  |
| Radial | 0.53 (0.51-0.55) | <0.001 | 0.61 (0.59-0.62) | <0.001 |
| Others | 0.85 (0.79-0.91) | <0.001 | 0.76 (0.72-0.80) | <0.001 |
| Mechanical circulatory support during PCI |  |  |  |  |
| IABP | 2.89 (2.77-3.01) | <0.001 | 5.31 (5.15-5.48) | <0.001 |
| VA-ECMO | 5.67 (5.34-6.02) | <0.001 | 4.69 (4.44-4.96) | <0.001 |
| PVAD | 2.71 (2.48-2.96) | <0.001 | 4.21 (3.90-4.55) | <0.001 |
| Drug-eluting stent use | 0.63 (0.60-0.65) | <0.001 | 0.74 (0.72-0.77) | <0.001 |
| Bare-metal stent use | 1.17 (0.90-1.52) | 0.24 | 2.03 (1.73-2.38) | <0.001 |
| Drug-coated balloon use | 0.62 (0.58-0.65) | <0.001 | 0.66 (0.63-0.68) | <0.001 |
| Training facilities (vs. non-training facilities) | 0.81 (0.76-0.86) | <0.001 | 0.89 (0.86-0.93) | <0.001 |
| After the 2020 policy change for RA use (Years 2021-2023) | 1.13 (1.05-1.20) | <0.001 | 1.01 (0.96-1.06) | 0.72 |
| Interaction (facility type × time period) | 0.98 (0.90-1.06) | 0.55 | 1.08 (1.02-1.14) | 0.006 |

CABG, coronary artery bypass grafting; CI, confidence interval; IABP, intra-aortic balloon pump; NSTEMI, non-ST-segment elevation myocardial infarction; PCI, percutaneous coronary intervention; PVAD, percutaneous ventricular assist device; SD, standard deviation; STEMI, ST-segment elevation myocardial infarction; VA-ECMO, venoarterial extracorporeal membrane oxygenation.

**Supplemental Table 2.** Adjusted odds ratios for in-hospital mortality among patients undergoing PCI with RA after the 2020 RA facility criteria revision.

|  | **In-hospital death** |  |
| --- | --- | --- |
| **Variables** | **Odds ratio (95% CI)** | **P value** |
| Age, per 1 year | 1.06 (1.05-1.07) | <0.001 |
| Male | 0.90 (0.74-1.1) | 0.32 |
| Hypertension | 0.92 (0.72-1.17) | 0.48 |
| Diabetes | 1.27 (1.04-1.55) | 0.017 |
| Hyperlipidemia | 0.69 (0.56-0.84) | <0.001 |
| Dialysis | 1.86 (1.46-2.35) | <0.001 |
| Chronic lung disease | 1.06 (0.67-1.67) | 0.80 |
| Peripheral arterial disease | 1.52 (1.20-1.92) | <0.001 |
| Prior PCI | 0.93 (0.75-1.16) | 0.52 |
| Prior CABG | 0.99 (0.71-1.40) | 0.96 |
| Prior myocardial infarction | 1.22 (0.97-1.53) | 0.097 |
| Prior heart failure | 1.73 (1.42-2.11) | <0.001 |
| Elective PCI | 0.60 (0.46-0.78) | <0.001 |
| Acute coronary syndrome | 2.89 (2.16-3.86) | <0.001 |
| Cardiac arrest within 24 hours | 2.65 (1.76-3.98) | <0.001 |
| Cardiogenic shock within 24 hours | 2.22 (1.60-3.08) | <0.001 |
| Acute heart failure within 24 hours | 1.46 (1.09-1.95) | 0.012 |
| Anginal symptom within 1 month | 0.62 (0.48-0.82) | <0.001 |
| Pre-procedural clopidogrel | 0.75 (0.54-1.03) | 0.077 |
| Pre-procedural potent P2Y12 inhibitors (ticagrelor or prasugrel) | 0.89 (0.66-1.19) | 0.43 |
| Pre-procedural anticoagulants | 1.15 (0.85-1.55) | 0.37 |
| Arterial access site (vs. femoral) |  |  |
| Radial | 0.71 (0.57-0.89) | 0.002 |
| Others | 1.24 (0.90-1.70) | 0.20 |
| Mechanical circulatory support during PCI |  |  |
| IABP | 4.63 (3.71-5.77) | <0.001 |
| VA-ECMO | 8.29 (5.86-11.72) | <0.001 |
| PVAD | 4.45 (3.03-6.55) | <0.001 |
| Drug-eluting stent use | 0.78 (0.60-1.00) | 0.052 |
| Bare-metal stent use | 1.35 (0.32-5.74) | 0.69 |
| Drug-coated balloon use | 0.60 (0.47-0.77) | <0.001 |
| Training facilities (vs. non-training facilities) | 0.66 (0.50-0.89) | 0.005 |

CABG, coronary artery bypass grafting; CI, confidence interval; IABP, intra-aortic balloon pump; NSTEMI, non-ST-segment elevation myocardial infarction; PCI, percutaneous coronary intervention; PVAD, percutaneous ventricular assist device; SD, standard deviation; STEMI, ST-segment elevation myocardial infarction; VA-ECMO, venoarterial extracorporeal membrane oxygenation.

**Supplemental Table 3.** In-hospital outcomes after PCI with RA at training and non-training facilities before and after the 2020 facility criteria revision.

|  | **Before the facility criteria revision** |  | **After the facility criteria revision** |  |  |
| --- | --- | --- | --- | --- | --- |
|  | 2019 | 2020 | 2021 | 2022 | 2023 |
| In-hospital death |  |  |  |  |  |
| Training | 1.5 (149/10249) | 1.5 (149/10115) | 1.4 (164/11554) | 1.6 (186/11287) | 1.6 (174/10608) |
| Non-training | - | - | 1.2 (7/594) | 2.1 (24/1163) | 2.6 (41/1556) |
| PCI-related myocardial infarction |  |  |  |  |  |
| Training | 1.5 (149/10249) | 2.0 (199/10115) | 1.5 (175/11554) | 1.9 (218/11287) | 1.7 (18010608) |
| Non-training | - | - | 1.7 (10/594) | 1.8 (21/1163) | 1.8 (28/1556) |
| Cardiac tamponade |  |  |  |  |  |
| Training | 0.5 (47/10249) | 0.3 (35/10115) | 0.3 (33/11554) | 0.4 (42/11287) | 0.4 (40/10608) |
| Non-training | - | - | 0 (0/594) | 0.5 (6/1163) | 0.4 (7/1556) |
| Cardiogenic shock requiring mechanical and/or inotropic support |  |  |  |  |  |
| Training | 1.4 (143/10249) | 1.8 (178/10115) | 1.5 (169/11554) | 1.5 (174/11287) | 1.6 (167/10608) |
| Non-training | - | - | 1.3 (8/594) | 2.0 (23/1163) | 1.8 (28/1556) |
| Stent thrombosis |  |  |  |  |  |
| Training | 0.1 (10/10249) | 0.1 (9/10115) | 0.04 (5/11554) | 0.1 (9/11287) | 0.1 (11/10608) |
| Non-training | - | - | 0 (0/594) | 0 (0/1163) | 0 (0/1556) |
| Requirement for emergency surgery |  |  |  |  |  |
| Training | 0.2 (25/10249) | 0.1 (10/10115) | 0.2 (20/11554) | 0.2 (21/11287) | 0.2 (17/10608) |
| Non-training | - | - | 0.2 (1/594) | 0.3 (3/1163) | 0.2 (3/1556) |
| Bleeding requiring blood transfusion |  |  |  |  |  |
| Training | 0.8 (84/10249) | 1.0 (103/10115) | 0.9 (109/11554) | 0.9 (97/11287) | 1.1 (118/10608) |
| Non-training | - | - | 1.5 (9/594) | 1.1 (13/1163) | 1.0 (16/1556) |
| Access site bleeding |  |  |  |  |  |
| Training | 0.5 (51/10249) | 0.6 (63/10115) | 0.6 (73/11554) | 0.5 (55/11287) | 0.7 (73/10608) |
| Non-training | - | - | 0.8(5/594) | 0.9 (10/1163) | 0.6 (9/1556) |
| Non-access site bleeding |  |  |  |  |  |
| Training | 0.3 (34/10249) | 0.4 (41/10115) | 0.3 (38/11554) | 0.4 (44/11287) | 0.5 (49/10608) |
| Non-training | - | - | 0.7 (4/594) | 0.3 (4/1163) | 0.4 (7/1556) |
| In-hospital death or any complication |  |  |  |  |  |
| Training | 4.6 (473/10249) | 5.4 (543/10115) | 4.8 (552/11554) | 5.4 (604/11287) | 5.2 (549/10608) |
| Non-training | - | - | 5.1 (30/594) | 6.0 (70/1163) | 5.9 (92/1556) |

Data are expressed as No. (%) of patients. Until April 2020, PCI operators were not permitted to perform rotational atherectomy during PCI at non-training facilities in Japan. Annual hospital PCI volumes ≥200 cases and on-site surgical backup are mandatory for training facilities.

PCI, percutaneous coronary intervention.
